# Supplementary material for: Imperfect two-dimensional topological insulator field-effect transistors
Source: Nat Commun. 2017 Jan 20;8:14184. doi: 10.1038/ncomms14184 (PMC5263869; doi:10.1038/ncomms14184)
Supplement: Supplementary Information — Supplementary Figures [file ncomms14184-s1.pdf]

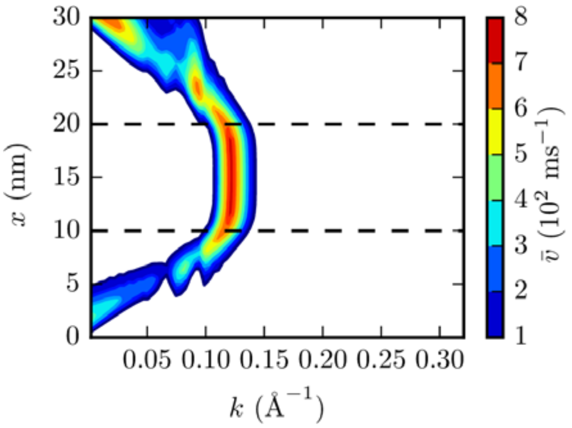

Supplementary Figure 1: Net velocity of electrons with opposite momentum $\bar{v}(x,k)=v(k)(\ f(x,k)-f(x,-k)\ )$ for V_{gs}=0.5 V and V_{ds}=0.1 V. Current (integral of the net velocity over *k*) is continuous as a function of *x*. Comparing to the Boltzmann distribution shown in Fig. 3b from the main manuscript, we can see that the current is carried by those states where the distribution makes a transition from 0 to 1.
